# Rates of Bilateral Mastectomy in Patients With Early-Stage Breast Cancer

**DOI:** 10.1001/jamanetworkopen.2022.51348

**Published:** 2023-01-18

**Authors:** Marie Fefferman, Kyra Nicholson, Kristine Kuchta, Catherine Pesce, Katherine Kopkash, Katharine Yao

**Affiliations:** 1 Department of Surgery, University of Chicago, Chicago, Illinois; 2 Biostatistical Core, NorthShore University HealthSystem Research Institute, Evanston, Illinois; 3 Department of Surgery, NorthShore University HealthSystem, Evanston Hospital, Evanston, Illinois

## Abstract

This cohort study builds on previous research from the National Cancer Database to assess whether rates of bilateral mastectomy continue to increase.

## Introduction

Studies have demonstrated increasing rates of bilateral mastectomy (BM) since the late 1990s.^1,2,3^ The first study reported increasing rates of BM from 1998 to 2003 and subsequent studies examined rates up to 2007 and then 2011.^1,2,3^ A study showed increasing rates of BM from 1998 to 2007.^2^ The aim of this study was to assess whether BM rates are still increasing.

## Methods

We examined rates of BM, unilateral mastectomy (UM), and breast-conserving surgery (BCS) for patients older than 18 years with stage 0 to II breast cancer from January 2008 to December 2020, using the National Cancer Database. Women undergoing neoadjuvant therapy and women with bilateral breast cancer or a history of breast cancer were excluded. Rates of BM were stratified by patient age by decade, race and ethnicity, pathologic stage, insurance status, location, and facility type. Data on race and ethnicity were collected as potential factors associated with BM. Differences in BM rates from 2013 to 2020 were compared using an interaction term with year in linear regression models. All statistical analysis was performed with SAS, version 9.4 (SAS Institute Inc). Statistical tests were 2-tailed, and *P* < .05 was considered significant. This study was deemed exempt the NorthShore University HealthSystem Institutional Review Board because all data were deidentified. This study followed the STROBE reporting guideline for cohort studies.

## Results

**Figure 1.**
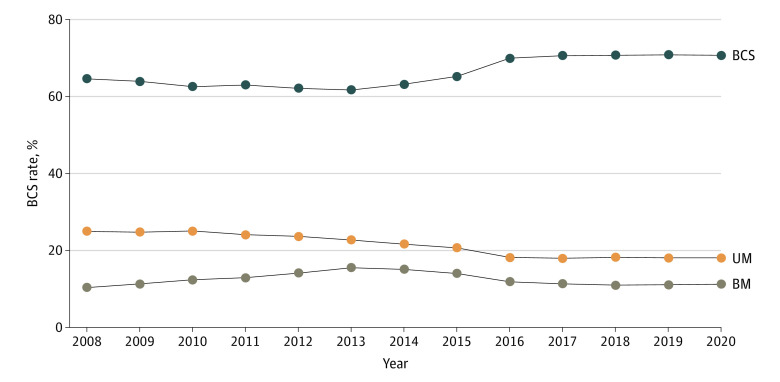
Rates of Breast Conserving Surgery (BCS), Unilateral Mastectomy (UM), and Bilateral Mastectomy (BM) From 2008 to 2020

In 988 666 patients in this study, the median age was 61 years (range, 18-90 years). Cancer was stage 0 in 196 672 patients (19.9%), stage I in 510 822 (51.7%), and stage II in 281 172 (28.4%). Of 988 666 patients, 656 836 (66.4%) underwent BCS, 208 010 (21.0%) underwent UM, and 123 820 (12.5%) underwent BM. Rates of BCS went from 64.6% in 2008 to 61.7% in 2013 and then increased to 70.7% in 2020 (Figure 1), UM rates decreased from 25.0% in 2008 to 18.1% in 2020, and BM rates went from 10.4% in 2008 to 15.6% in 2013 to 11.3% in 2020.

**Figure 2.**
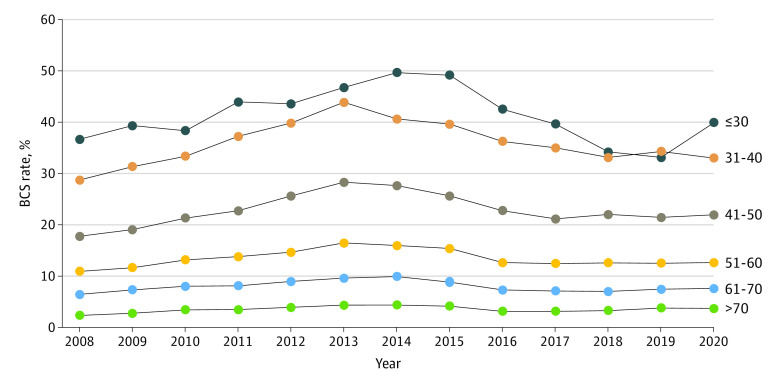
Bilateral Mastectomy Rates Stratified by Age Group From 2008 to 2020 BCS indicates breast conserving surgery.

The decrease in BM from 2013 to 2020 was noted across all age groups; however, younger patients showed a larger decrease than older patients (interaction *P* < .001) (Figure 2). Among women aged 30 years or younger, 49.7% underwent BM in 2014 vs 39.9% in 2020. For women aged 31 to 40 years, 43.9% underwent BM in 2013 vs 33.0% in 2020 (Figure 2). There was a decrease in the proportion of women undergoing BM starting in 2013 for all races and ethnicities, tumor stages, locations, facility types, and insurance statuses. Interaction between groups was significant only for geographic location and pathologic stage.

## Discussion

Rates of BM increased starting in 2008; however, the BM rates started to decrease in 2013, continued to do so until 2016, and then stabilized (Figure 1). Rates of BM in 2020 vs 2008 were similar (11.3% vs 10.4%). Rates were decreasing shortly after many of the studies on BM trends were published but before publication of the guidelines on BM.^1,2,3,4,5^ These findings are supported by a recent study that reported a stabilization of BM rates from 2013 to 2017.^6^ Data examining surgical trends often lag several years, making it difficult to note surgical trends in real time. The decrease in BM rates may reflect surgeon efforts based on the increasing number of publications on BM trends. This study has limitations, including a lack of information on germline mutations and family history, which may influence the decision to pursue BM. The generalizability of this study is limited because the data are not population-based. We will continue to monitor BM trends to determine the outcomes associated with the COVID-19 pandemic.

## Notes

Supplement.
Data Sharing Statement
