# Errors in Axis Labels in Figures

**DOI:** 10.1001/jamanetworkopen.2023.6719

**Published:** 2023-03-20

In the Research Letter titled “Rates of Bilateral Mastectomy in Patients With Early-Stage Breast Cancer,”^1^ published January 18, 2023, there were errors in the labels of the y-axes in Figure 1 and Figure 2. Both axes are labeled “BCS rate, %.” The y-axis label for Figure 1 should have read “Surgery rate, %.” The y-axis for Figure 2 should have read "BM rate, %." This article has been corrected.^1^
