# Supplementary material for: Rates of Bilateral Mastectomy in Patients With Early-Stage Breast Cancer
Source: JAMA Netw Open. 2023 Jan 18;6(1):e2251348. doi: 10.1001/jamanetworkopen.2022.51348 (PMC9857138; doi:10.1001/jamanetworkopen.2022.51348)
Supplement: Supplement. — Data Sharing Statement [file jamanetwopen-e2251348-s001.pdf]

Data Sharing Statement

Fefferman. Rates of Bilateral Mastectomy in Patients With Early-Stage Breast Cancer. *JAMA Netw Open*. Published January 18, 2023. doi:10.1001/jamanetworkopen.2022.51348

Data
Data available: No

Additional Information
Explanation for why data not available: The data is already available through the National Cancer Database (NCDB).
